# Evaluation of Connexin 43 Redistribution and Endocytosis in Astrocytes Subjected to Ischemia/Reperfusion or Oxygen-Glucose Deprivation and Reoxygenation

**DOI:** 10.1155/2017/5064683

**Published:** 2017-03-23

**Authors:** Hongyan Xie, Yu Cui, Shuai Hou, Juan Wang, Jing Miao, Fang Deng, Jiachun Feng

**Affiliations:** ^1^Department of Neurology, Affiliated Hospital of Taishan Medical University, Tai'an 271000, China; ^2^Department of Neurosurgery, Affiliated Hospital of Taishan Medical University, Tai'an 271000, China; ^3^Department of Neurology, The First Hospital of Jilin University, Changchun 130021, China

## Abstract

Connexin 43 (Cx43) is the major component protein in astrocytic gap junction communication. Recent studies have shown the cellular processes of gap junction internalization and degradation, but many details remain unknown. This study investigated the distribution of Cx43 and its mechanism after ischemic insult. Astrocyte culture system and a model of ischemia/reperfusion (IR) or oxygen-glucose deprivation and reoxygenation (OGDR) were established. Cx43 distribution was observed by laser scanning confocal microscopy under different cultivation conditions. Western blot and RT-PCR assays were applied to quantify Cx43 and MAPRE1 (microtubule-associated protein RP/EB family member 1) expression at different time points. The total number of Cx43 was unchanged in the normal and IR/OGDR groups, but Cx43 particles in the cytoplasm of the IR/OGDR group were significantly greater than that of the normal group. Particles in the cytoplasm were significantly fewer after endocytosis was blocked by dynasore. There was no difference among the groups at each time point regarding protein or gene expression of MAPRE1. We concluded that internalization of Cx43 into the cytoplasm occurred during ischemia, which was partially mediated through endocytosis, not by the change of Cx43 quantity. Moreover, internalization was not related to microtubule transport.

## 1. Introduction

Stroke is a leading cause of death and long-term disability in humans. In central nervous system, astrocytes have diverse and important roles in many aspects of ischemic brain damage [1]. They form a glial network and communicate through gap junctions (GJs), allowing passage of ions and small metabolites among adjacent cells.

Vertebrate GJs consist of protein subunits termed connexins (Cx) [2]. Cx43 is the only connexin expressed by cultured astrocytes that was shown firstly by Giaume and Dermietzel et al. in 1991 [3, 4]; it is endogenously expressed in at least 35 tissues and cell types and is a major component protein in astrocytic GJs [5–7]. Since astrocytes are deemed an important participant in ischemia, their quantity and alteration have recently been the focus of research [8]. Exploring such features and the underlying molecular mechanisms of Cx43 by using an in vitro ischemic model may help to determine therapeutic target(s) in ischemia.

The short half-life of Cx proteins (1–5 h) [9] indicates GJ transport should be a dynamic progress. Furthermore, the relatively rapid rate of turnover in astrocytes is essential for Cx43-mediated GJ intercellular communication [10]. Modulation of the synthesis and degradation rate of Cx43 may be an important way to control the level of GJs under various conditions [6, 11]; regulation of GJs and Cx43 in astrocytes may answer to various stimuli like ischemia. Elevated levels of Cx43 immunoreactivity in astrocytic processes and cell bodies associated with brain ischemia have been demonstrated, and redistribution of Cx43 in myocardia answers to myocardial ischemia [12, 13].

Shinotsuka et al. [14] reported that astrocytes provided protection against tissue damage by way of their GJ-mediated intercellular network after oxygen and glucose deficiency. It is beneficial to study whether protective effects of astrocytes are mediated via Cx43 redistribution, and we focused on Cx43 redistribution and used carbenoxolone (CBX) to block GJs in this study. CBX, a nonselective blocker of connexin and pannexin hemichannels [15], has been widely used to block GJs in several systems and inhibits cell death [16].

The balance between synthesis and degradation of a protein determines its steady-state level. Some evidences showed that Cx transport was mediated partially by microtubules that are intrinsically polar: the plus-end is the fast-growing end and the minus-end is stable or serves as the site of disassembly [17]. Key to this process appears to be microtubule plus-end tracking proteins (+TIPs) like microtubule-associated protein RP/EB family member 1 (MAPRE1) and its homologues [18]; changes in MAPRE1 may reflect the formation, transport, and decomposition process of the microtubule. Thus, we observed not only microtubule transport by detecting changes of MAPRE1 gene expression and protein quantity, under normal conditions, but also conditions of ischemia/reperfusion (IR) or oxygen-glucose deprivation and reoxygenation (OGDR).

GJs are retrieved from the cell membrane to the cytoplasm for degradation [9]. However, whether this progress involves endocytic machinery needs to be determined [9]. There are approximately 10 different endocytic pathways coexisting in mammalian cells, and the majority of them are regulated by dynamin [19, 20]. Dynamin is a member of a superfamily of GTPases, which can pinch off invaginated membrane pits. Dynasore is a noncompetitive inhibitor of dynamin GTPase activity and blocks dynamin-dependent endocytosis in cells [21]. In the present study, Cx43 distribution was observed under IR/OGDR condition by using CBX or dynasore.

## 2. Materials and Methods

### 2.1. Astrocyte Cultures

The experiments were approved by the Experimental Animal Research Committee of Jilin University and were performed according to the Guidelines for Animal Experimentation of Jilin University. Primary astrocyte cultures were prepared from the cortex of 1-day-old Wistar rats. Rats were decapitated, and the cortices were dissected and transferred to cold D-Hanks solution. The tissues were incubated in phosphate-buffered saline (PBS) containing 0.125% trypsin for 10 min at 37°C. The reaction was terminated by Dulbecco's modified Eagle's medium/nutrient mixture F-12 containing 20% fetal bovine serum (FBS; GIBCO, Grand Island, USA). The tissues passed through a 40 *μ*m cell strainer. Cells were centrifuged at 1500 rpm for 10 min. The resulting cell pellets were resuspended and seeded into culture flasks at a concentration of 1 × 10^5^ cells/cm^2^ in culture dishes. The medium was changed 2x/week. When astrocytes in flasks reached confluence, they were separated into 2 dishes for further culture for 3-4 weeks. The final cultures containing >95% astrocytes and <5% microglia were verified for the following experiments.

### 2.2. In Vitro Model of Ischemia/Reperfusion (IR) or Oxygen-Glucose Deprivation and Reoxygenation (OGDR)

An in vitro model of IR/OGDR was made by incubating astrocyte cells in glucose-free Earle's balanced salt solution and oxygen deprivation media (bubbled with 5% CO_2_/94.9% N_2_/0.1% O_2_ for 30 min). Then reperfusion was initiated in normal media (DMEM/F12 containing 10% FBS) at 37°C in 5% CO2/95% air atmosphere at time points 0.5, 1, 3, 6, 12, 24, and 72 h.

### 2.3. Cell Survival Assessment

Cell survival was assessed by MTT (3-[4,5-dimethylthiazol-2-yl]-2,5-diphenyltetrazolium bromide) assay. Cells were cultured in 96-well plates at a density of 10,000 cells/well. After a 36 h treatment with various concentrations of CBX (5 mM, 1 mM, 500 *μ*M, 200 *μ*M, 100 *μ*M, 50 *μ*M, 20 *μ*M, and 10 *μ*M), 20 *μ*L of MTT solution (5 mg/mL) was added to each well and further incubated for 4 h. The MTT solution was aspirated gently. DMSO (150 *μ*L/well) was added to the wells. The cells were incubated with shaking for 10 min. The optical density of the supernatant was read at 570 nm (reference wavelength, 630 nm) using a microplate reader (Bio-Rad, USA). Absorbance was normalized to the untreated control cultures, which represented 100% viability. The viability rate was calculated as viability: % = mean absorbance of sample/mean absorbance of control × 100%. All data presented in this study were obtained from 5 independent experiments.

### 2.4. Immunofluorescence Staining

Cells were fixed in −20°C cold methanol. After washing in PBS, cells were blocked with 10% nonimmune goat serum (Sigma, St. Louis, USA) for 30 min at room temperature and incubated with 0.1% (v/v) Triton X-100 (Sigma, St. Louis, USA) for 5 min. The cells were serially incubated with primary antibodies at 4°C overnight. Control experiments were conducted under identical conditions, except that primary antibodies were replaced by PBS. Coverslips were incubated with the corresponding secondary antibody for 45 min. The following primary antibodies were used: rabbit anti-Cx43 (1 : 1000, Abcam, Cambridge, UK), mouse anti-glial fibrillary acidic protein (GFAP, 1 : 500; Boster, Wuhan, China), and rabbit anti-MAPRE1 (1 : 500, Abcam, Cambridge, UK). Secondary antibodies consisted of a 1 : 500 dilution of donkey anti-rabbit IgG conjugated to fluorescein isothiocyanate (FITC) (1 : 250, ZSGB-BIO, Beijing, China) and goat anti-mouse IgG conjugated to FITC (1 : 250, ZSGB-BIO, Beijing, China). After incubation, Hoechst 33342 (Sigma, St. Louis, USA) was added; incubation time was 20 min. All coverslips were observed under a laser-scanning confocal microscope (Olympus, Japan).

To evaluate changes in Cx43 distribution, we selected time points at which cell injury was observed to be the most severe, using MTT colorimetry. The cultures were divided into the following groups: normal culture, IR/OGDR saline, and IR/GDR with carbenoxolone (CBX) intervention. Observations were conducted using a confocal laser-scanning microscope. After blocking endocytosis with dynasore, the change in redistribution of Cx43 was observed under the confocal laser-scanning microscope. The relative fluorescence intensity of the cell and nuclei in each group was measured by Image-Pro Plus 7.0 Software, to provide semiquantitative analysis.

### 2.5. Western Blot Analyses

The methods described by Bradford (1976) were employed to detect the total protein concentration of cells. Protein homogenates of astrocyte cultures were subjected to SDS-polyacrylamide gel electrophoresis and then transferred to a polyvinylidene fluoride (PVDF) membrane. After blocking, each membrane was incubated overnight at 4°C with rabbit anti-Cx43 (1 : 5000, Abcam, Cambridge, UK), anti-MAPRE1 antibody (1 : 500, Abcam, Cambridge, UK), or anti-*β*-actin antibody (1 : 10,000, Sigma, St. Louis, USA). The membrane was then incubated with an IgG alkaline phosphatase-conjugated antibody (1 : 10,000, Sigma, St. Louis, USA) for one hour at 37°C. The membrane was washed, and MAPRE1 and *β*-actin were detected using a BCIP/NBT substrate system (Sigma, St. Louis, USA). The optical density of each protein band was measured with NIH Image J software and normalized as to the optical density of the corresponding *β*-actin. All samples were made in triplicate.

### 2.6. RNA Extraction and RT-PCR Assay

Cells were collected into a 1.5 mL Eppendorf tube by repeatedly pipetting with 1 mL Trizol. Added to this was 0.2 mL chloroform. These were vigorously oscillated and then placed at room temperature for 3 min. The water phase was transferred to a new tube after centrifugation and kept at −20°C for 20 min with the same volume of isopropyl alcohol. The RNA precipitate was collected after centrifugation. One mL of 75% ethanol was used to wash the RNA precipitate, which was placed at room temperature for about 5–10 min; RNase-free water was used to dissolve the RNA, and then it was preserved at −70°C. The amount of total RNA was measured using a NanoDrop 2000/2000c (Thermo Scientific, Dreieich, Germany). All samples were made in triplicate.

For each sample, total RNA was reversely transcribed into cDNA with a SuperScript II Reverse Transcriptase Kit (Invitrogen, USA). The reaction was allowed for 5 min at 30°C, 60 min at 42°C, and 15 min at 72°C. RT-PCR was performed using 20 *μ*L of a mixture composed of 10 *μ*L SYBR, 0.4 *μ*L S-Primer, 4 *μ*L AS-Primer, 0.4 *μ*L ROX, 1 *μ*L cDNA, and 7.8 *μ*L ddH_2_O (Please describe volume correctly). The reaction was performed in the IQ5 iCycler (Bio-Rad, Munchen, Germany) using the following 3-step amplification protocol: 30 s at 95°C (enzyme activation), 40 cycles of 5 s at 95°C (denaturation), 34 s at 61°C (annealing), and 15 s at 95°C and 60 s at 60°C (extension). RT-PCR products were resolved in 1.2% agarose by gel electrophoresis. Cycle threshold (Ct) values were statistically analyzed using REST Software [22]. The sequences of the primers we used are as follows: rat MAPRE1 sense, 5′-AAG AGG ATG GGC GTT GAC A -3′; and antisense, 5′-AAG GAG CCA CTG CCG TTT C-3′; and rat *β*-actin sense, 5′-GAG GCC CCT CTG AAC CCT AA-3′; and antisense, 5′-ACC AGA GGC ATA CAG GGA CAA-3′.

### 2.7. Statistical Analysis

The data were analyzed using SPSS19.0 software. All data are shown as mean ± standard deviation. Comparisons among 2 or more groups were performed by means of an independent-samples* t*-test and analysis of variance, followed by Fisher's partial least-squares difference. Differences with a *P* value ≤ 0.05 were considered statistically significant. The graphs were drawn by GraphPad Prism 6.0 software.

## 3. Results

### 3.1. Determination of CBX Concentration and Astrocytes in Response to IR/OGDR

The MTT colorimetric method was applied to analyze the cytotoxicity of CBX and to determine the optimal concentration. No obvious toxic effect on the survival of astrocytes was observed when CBX was at the concentration of 20 *μ*mol/L. Therefore, 20 *μ*mol/L of CBX was used in this study. Cultured astrocytes were subjected to oxygen-glucose deprivation for 2 h and then to normal supplements of oxygen and glucose at 0.5, 1, 3, 6, 12, 24, or 72 h. Astrocytes were most severely damaged, and viability was lowest 6 h after reperfusion and then gradually improved and returned to nearly normal level at 72 h. Therefore, the time point 6 h after reperfusion was chosen for observation during the follow-up study.

### 3.2. Influence of IR/OGDR on Redistribution of Cx43

**Figure 1:**
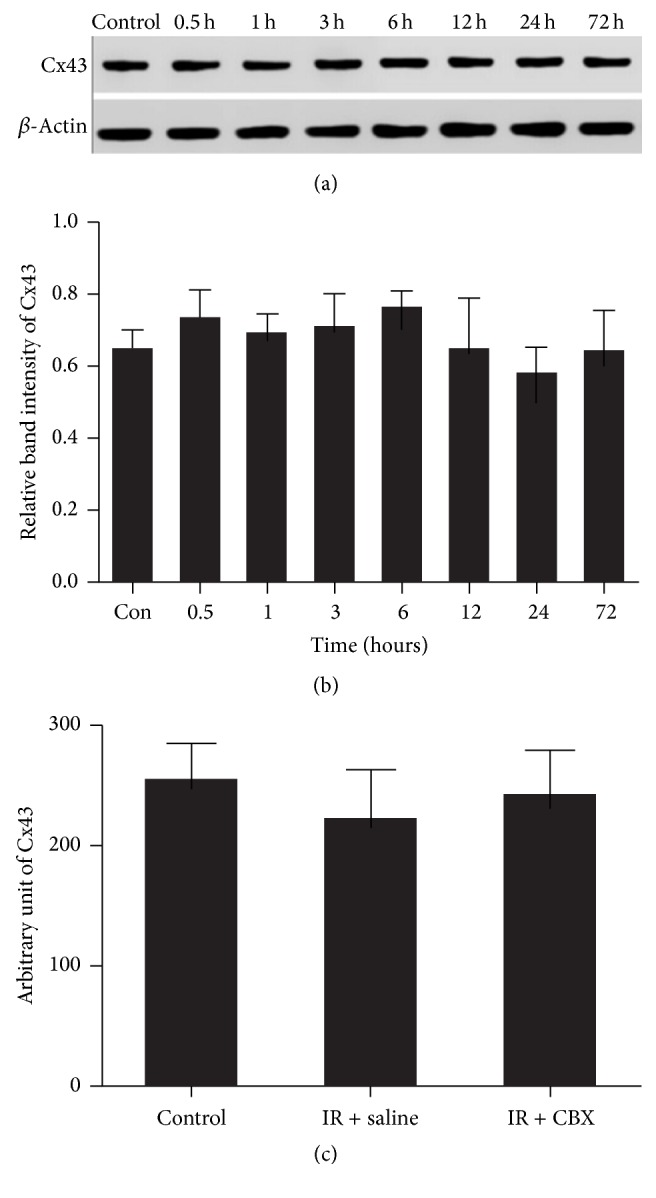
Representative western blot of Cx43 in the normal and at different time points of IR/OGDR groups. (a) The first stripe was Cx43 protein; the other was *β*-actin as the internal stripe. (b) Cx43 protein relative expression. There was no significant difference among the groups at each time point (*P* = 0.307). (c) Representative graph of fluorescence intensity value of Cx43 in normal and IR/OGDR 6 h (saline/CBX) groups. There was no significant difference among the three groups (*P* = 0.208).

We made the quantitative analysis of Cx43 in the normal and at different time points of IR/OGDR groups. As shown in Figures 1(a) and 1(b), the total amount of Cx43 was unchanged under normal and IR/OGDR conditions (*P* = 0.307). We analyzed the fluorescence intensity of Cx43 in astrocytes by Image-Pro Plus 7.0 Software and drew the graph by GraphPad Prism 6.0 software. As shown in Figure 1(c), the total level of Cx43 of astrocytes was unchanged among groups; only distribution occurred. This was consistent with the conclusion of Figures 1(a) and 1(b).

**Figure 2:**
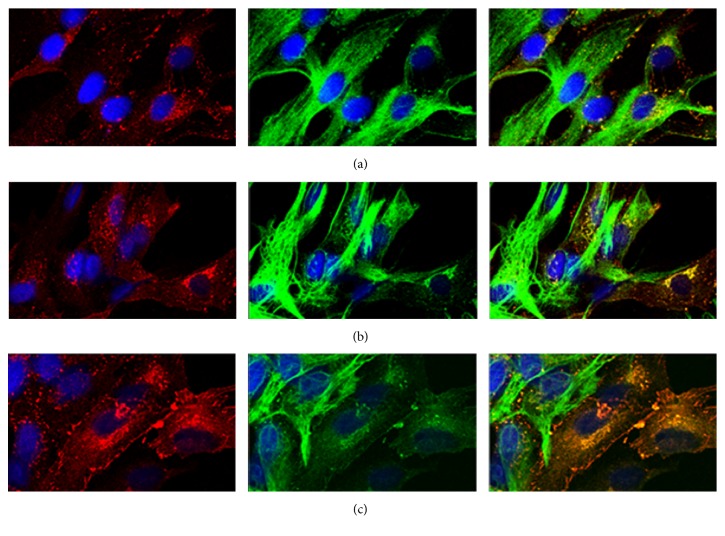
Representative confocal images of Cx43 distribution under different conditions at 6 h after reperfusion (red, Cx43; green, GFAP; blue, Hoechst 33342; magnification was 400x, resp.). (a) Under normal cultivation conditions, Cx43 was mainly distributed in the cytoplasmic membrane, and its density was higher in the adjacent cell membrane joint. A small amount of scattered Cx43 has been circulated in the cytoplasm with a few particles distributed in the nucleus. (b) Under IR/OGDR conditions with physiological saline, Cx43 was distributed in the membrane, cytoplasm, and nucleus, with the highest content in the cytoplasm. These vesicles of different sizes and shapes were observed in the cytoplasm. The total number of particles of this group was almost the same as that of the normal group (*P* = 0.761), but the cytoplasm particles of this group were significantly more than those of the normal group (*P* = 0.019). (c) Under IR/OGDR conditions with CBX intervention, Cx43 was distributed in the membrane, cytoplasm, and nucleus but mainly in the cytoplasm. There was no significant difference between the CBX intervention and saline control group (*P* = 0.217).

We examined the distribution of Cx43 with a laser-scanning confocal microscope under normal and IR/OGDR conditions. As shown in Figures 2(a) and 2(b), it was found that there was no difference in the total amount of Cx43 (*P* = 0.761) between the normal and IR/OGDR groups; however, the cytoplasm particles of the IR/OGDR group were significantly greater in number than that of the normal group (*P* = 0.019). Furthermore, some vesicles of different sizes and shapes appeared in the cytoplasm of the IR/OGDR group, which were obviously distinct from the endoplasmic reticulum and Golgi apparatus transport vesicles in the normal group. Under IR/OGDR conditions with CBX intervention, which was shown in Figure 2(c), Cx43 redistribution was not affected (*P* = 0.217) compared with the saline control group.

### 3.3. Effect of Dynasore on Redistribution of Cx43

**Figure 3:**
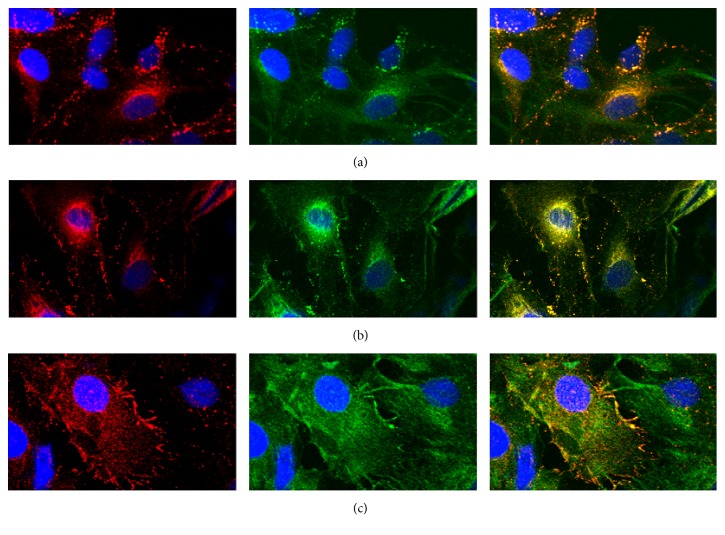
Representative confocal images showed Cx43 distribution under IR/OGDR conditions at 6 h after reperfusion (red, Cx43; green, GFAP; blue, Hoechst 33342; magnification was 400x, resp.). (a) Under normal cultivation conditions, the distribution of Cx43 is similar to that in Figure 1(a). (b) Under IR/OGDR conditions with DMSO (as the medium to dissolve dynasore), the distribution of Cx43 was almost the same as that in Figure 2(b). (c) Under IR/OGDR conditions with dynasore, Cx43 was distributed in the membrane, cytoplasm, and nucleus, with the highest content in the cytoplasm. The particles in the cytoplasm of this group were significantly fewer than those of the DMSO control group (*P* = 0.021), and the number of particles transported into cytoplasm was reduced by 29.2%.

To block endocytosis, dynasore was used. In addition, DMSO, as a universal solvent to dissolve dynasore, was selected as the control group to rule out its influence. According to the literature, we knew that the final working concentration for in vivo experiments was 80 *μ*mol/L (0.2% DMSO final), which typically resulted in a greater than 90% blockage in endocytosis [21]. Therefore, we selected 80 *μ*mol/L as the working concentration of dynasore. As shown in Figures 3(a) and 3(c), we found that Cx43 still moved from the cell membrane to the cytoplasm when endocytosis was blocked with dynasore. As depicted in Figures 3(b) and 3(c), the cytoplasm particles of the group under IR/OGDR conditions with dynasore were significantly fewer than those in the DMSO control group (*P* = 0.021), and the number of particles transported into the cytoplasm was reduced by 29.2%.

### 3.4. No Significant Difference in MAPRE1 among All Groups under IR/OGDR Conditions

**Figure 4:**
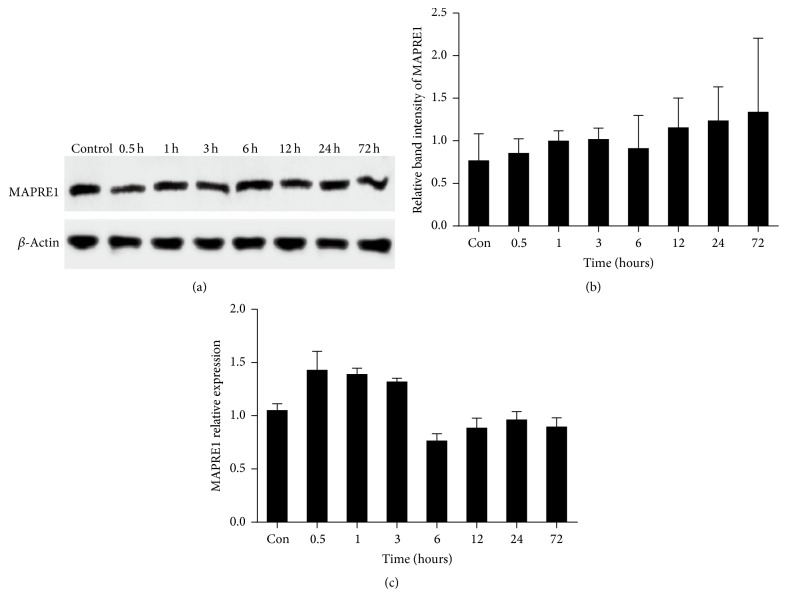
Representative western blot and RT-PCR assays of MAPRE1. (a) The first stripe was MAPRE1 protein; the other was *β*-actin as the internal stripe. (b) MAPRE1 protein relative expression. While there was no significant difference among the groups at each time point (*P* = 0.69), levels were lowest at IR/OGDR 6 h. (c) MAPRE1 gene expression. While there was no significant difference among the groups (*P* = 0.634), gene expression was least at IR/OGDR 6 h.

Western blot was applied for quantitative analysis of the MAPRE1 protein. There was no significant difference in the protein levels of MAPRE1 among the groups at different times (*P* = 0.69). Moreover, we analyzed the gene expression of MAPRE1 via RT-PCR, and no significant difference was observed among the groups at each time point (*P* = 0.634); however, its mRNA amount at 6 h was minimal (Figure 4). It is suggested that the transportability of microtubules was lowest at this time. These results showed that transport by microtubules may be reduced, indicating that Cx43 synthesized in the endoplasmic reticulum could not be transported efficiently to the cell membrane.

## 4. Discussion

In the present study, we showed that the total amount of Cx43 was unchanged under IR/OGDR conditions. However, some internalized vesicles that were different from endoplasmic reticulum or Golgi apparatus transport vesicles were observed in the cytoplasm of the IR/OGDR group. Moreover, CBX did not affect Cx43 redistribution. There was no statistical difference among the groups in protein levels or gene expression of MAPRE1. Particles in the cytoplasm in the dynasore intervention group were significantly fewer than in the control group under IR/OGDR conditions. We concluded that internalization of Cx43 into the cytoplasm occurred during ischemia, which was partially mediated through endocytosis, and not by a change in the number of Cx43 particles. Moreover, internalization was not related to microtubule transport.

Alterations in Cx43 expression and function are involved in the pathophysiology of some diseases, such as brain ischemia [23]. Nakase et al. [24] were the first to investigate the level of Cx43 expression in the human brain, finding that it increased under long-term ischemia. Downregulation of Cx43 significantly increased the survival of pyramidal neurons and improved cognitive impairment after middle cerebral artery occlusion (MCAO) [25]. However, Cx43 knockout mice showed a significantly increased brain stroke volume and enhanced apoptosis [26]. Orellana et al. showed that hypoxia-reoxygenation enhanced activity of Cx43 hemichannels and reduced gap junctional coupling of cortical astrocytes in culture, and these effects were potentiated by high as well as zero glucose during the hypoxic period [27]. Later, Orellana et al. also found that reoxygenation after 3 h hypoxia in high glucose induced transient astroglial permeabilization and reduction in intercellular communication via Cx43 based channels [28]. As the above conclusions were derived from different experimental systems, it is difficult to predict the roles of connexins in ischemia. Therefore, we used IR/OGDR model to mimic cerebral ischemia and observed the change of Cx43.

The present study showed that the total number of Cx43 was unchanged in the normal and IR/OGDR groups; however, some internalized vesicles were observed in the cytoplasm of the IR/OGDR group. Their size was smaller than that of organelles in the cytoplasm, which was observed roughly under a laser-scanning confocal microscope. The size of these vesicles could not be accurately measured due to the limitations of the experimental conditions, which required further experiments. Martinez and Sáez found that hypoxia/reoxygenation altered gap junctions and was associated with cellular redistribution of proteins, which was believed to constitute a lysosomal pathway for Cx43 degradation [29]. Similar to this report, we also found that Cx43 was transported into the cytoplasm and nuclei during ischemia, which was consistent with the fact that transfected Cx43 was predominantly localized in the cytosol and nucleus [30]. Moreover, it has been reported that Cx43 could bind to DNA, suggesting that Cx43 has distinct functions from its well-known involvement in GJ intercellular communication [31]. Orellana et al. [32] showed that chlorpromazine did not induce obvious cell shape changes in astrocyte cultures but it clearly changed the distribution of Cx43, and this distribution was based on changes in the state of microtubules and microfilament. In agreement with this viewpoint, we analyzed the distribution of Cx43 and unchanged expression of MAPRE1 under normal and IR/OGDR conditions. Alternatively, it might be explained that the functions of gap junction channels were regulated by the state of Cx43 and MAPRE1 but not by their quantity.

As the licorice derivative, CBX can block almost all types of GJs regardless of Cx composition, and it is well tolerated at even high doses [33]. We used CBX at 20 *μ*mol/L, which was confirmed by an MTT assay, consistent with a previous report [34]. Frantseva et al. [35] found that CBX was neuroprotective but did not significantly alter intrinsic neuronal characteristics. Also, CBX does not penetrate the blood-brain barrier [36]. Therefore, for the treatment group in the present study, we applied CBX to block GJs, to examine whether its neuroprotective role involved Cx43. The results showed that CBX treatment did not change Cx43 redistribution. This suggested that the quantity was changed by CBX, and it may invaginate into the cytoplasm to form utricle bubbles. Up to now, CBX is a well-known molecule that is able to inhibit some subtypes of gap junction proteins including Cx43 and gap-junction-related protein called pannexin-1 [37]. Moreover, CBX affects multiple targets, including voltage-gated calcium channels, intrinsic neuronal properties, and neurotransmitter release and acts as an anticonvulsant [38]. And thus it can be seen that CBX has a wide range of effects, which is also doomed to have many side effects such as cytotoxicity. Cytotoxicity of CBX on astrocytes was determined by performing the MTT assay. Relative to other concentrations of CBX, 20 *μ*mol/L did not cause cytotoxic effect on the survival of astrocytes. Meanwhile, this concentration of CBX could block gap junctional communication, Cx43 hemichannels, and pannexin 1 channels as well.

As the transport of Cx43 is mediated by microtubules, it is phosphorylated and oligomerized into hexameric structures termed connexon [39]. MAPRE1 functions in GJ formation, which promotes delivery of Cx to the cell-cell border [40]. Some studies have shown that microtubules were dispensable for the regulation of Cx43 [41]. Recent studies revealed that Cx43 modulated cytoskeletal proteins including MAPRE1, which are involved in process formation and migration of astrocytes. Besides, Stephan et al. found that MAPRE1 expression depended on the level of Cx43 expression by the method of immunocytochemistry and western blot [42]. In the present study, we showed that there was no difference in protein and gene expression of MAPRE1 after IR/OGDR at time points 0.5, 1, 3, 6, 12, 24, and 72 h. In agreement with this viewpoint, we also found that the total number of Cx43 was unchanged in the normal and IR/OGDR groups. Although the levels of protein and gene expression of MAPRE1 were not different after IR/OGDR at every time point examined, it could be explained that the integrity or redistribution of microtubules and microfilament might be more relevant to the trafficking of Cx43 containing vesicles. Thereby, this report offered the chance to gain further insights into the mechanisms by which astrocytes could achieve distribution of Cx43 after ischemic insult.

Entire or fragments of GJs internalized as double-membrane circular structures were traditionally called annular junctions [43]. A recent study revealed that annular junctions originated from preexisting gap junction plaques at cell-cell interfaces [44]. Gaietta et al. [45] showed that newly synthesized GJs are assembled from the outer edges of plaques, whereas aged channels were removed to the central core. These internalized GJs are large and structurally different from typical endocytic cargo [46] and are likely to be distinct from conventional endocytic processes, such as classical endosomes or phagosomes [6, 46]. Recent studies found that some dynamin was recruited to the endocytic pathway to abstrict invagination of vesicles during evolution [47]. Gumpert et al. [48, 49] reported clathrin-mediated endocytosis, the most extensive route involving dynamin. Our results showed that internalization was reduced partially by dynamin. This supported the role of dynamin in GJ internalization.

Evidence has shown that the rate of Cx43 degradation can be modulated at both the level of GJ endocytosis and postendocytic sorting of Cx to lysosomes [50]. The researchers provided molecular and mechanistic insights into GJ degradation, utilizing clathrin-mediated endocytosis components [9, 51]. Cx43 may function not only in early noticeable invagination, but also in the process of degradation of this structure. It is suggested that dynamin interferes with successive stages of the internalization process [52].

In this study, we analyzed changes in Cx43 and its related mechanism by using laser-scanning confocal microscopy, western blot, and RT-PCR. This is conducive to evaluate comprehensively the effect of Cx43 on the structure and function of the brain, to reveal the role of Cx43 in ischemic stroke, and to provide new ideas for treatment. On the basis of our observations of the changes of Cx43 after ischemia, we analyzed its mechanism from the aspects of microtubule transport and endocytosis for an in-depth understanding of the factors that influence Cx43 functional changes and provide a theoretical basis for selecting a method of clinical intervention. These results indicate that Cx43 internalization may be considered a novel therapeutic target.

The internalization of GJs is a rarely reported mechanism. Although we demonstrated that Cx43 internalization was partially associated with endocytosis, the mechanisms of Cx43 internalization remain to be elucidated. Increasing evidence indicates that the process may involve phosphorylation and ubiquitination or polypeptide domains that act as sorting signals during ischemia/hypoxia [7, 53, 54]. Further studies are necessary to identify all the pathways responsible for Cx43 internalization.

Under conditions of ischemia, the amount of Cx43 did not change, but internalization of Cx43 occurred. This internalization was partly mediated through endocytosis, in response to the ischemic insult. Furthermore, internalization was not related to microtubule transport. The involvement of Cx43 within astrocytes during ischemic insults warrants study of the protective roles of astrocytes and gap conjunctions and provides a therapeutic target for treatment of brain ischemia.
